# Complete genome sequence of *Paracoccus marcusii* phage vB_PmaS-R3 isolated from the South China Sea

**DOI:** 10.1186/s40793-015-0089-7

**Published:** 2015-11-10

**Authors:** Yongle Xu, Rui Zhang, Nianzhi Jiao

**Affiliations:** State Key Laboratory of Marine Environmental Science, Institute of Marine Microbes and Ecospheres, Xiamen University, Xiamen, China

**Keywords:** Short genome report, vB_PmaS-R3, *Paracoccus marcusii* phage, South China Sea, Genome sequence

## Abstract

*Paracoccus* spp. are isolated from both terrestrial and aquatic habitats, indicating their ubiquitous existence in the environment. Here we present the first phage isolated from this genus, vB_PmaS-R3, and its complete genome sequence. *Paracoccus* phage vB_PmaS-R3 is a siphophage isolated from the South China Sea. The genome sequence is 42,093 bp, with a G + C content of 56.36 %. Fifty-two open reading frames were predicted from the genome. The genome can mainly be divided into three regions: genes for DNA metabolism, regulatory genes and structure forming genes. Genes encoding DNA metabolism and structural proteins showed high sequence homology to corresponding genes of *Burkholderia* phage KL1 and *Pseudomonas* phage PA73. In addition, four gene transfer agent-like genes were found in the vB_PmaS-R3 genome. A putative L-alanoyl-D-glutamate peptidase was predicted as the endolysin. A *MazG* gene was found in the vB_PmaS-R3 genome, which indicates genomic adaption to the nutrient-limited marine environment.

**Electronic supplementary material:**

The online version of this article (doi:10.1186/s40793-015-0089-7) contains supplementary material, which is available to authorized users.

## Introduction

Viruses are the most abundant biological entities in the marine environment [1]. They mediate approximately 20 % mortality of the biomass in the sea per day, accelerate nutrient cycling rates and regulate the host community composition through host-specific infection and lysis of cells [1, 2]. Viruses also play an important role in host evolution through viral infection-mediated lateral gene transfer [3]. Metagenomic studies of several marine viral communities revealed vast genetic diversity and contained a large fraction of novel sequences [2, 4]. Studies on virus isolation, virus-host interaction and genomic analysis at the individual level can provide detailed information on the biological features of a virus and give a better understanding of its role in biogeochemical and evolutionary processes [5]. *Paracoccus* is an important genus in the *Rhodobacteraceae* family. *Paracoccus* spp. are widely distributed in both terrestrial and aquatic environments and frequently isolated from marine environments [6, 7]. Although there are many species identified in the *Paracoccus* genus, so far there is no report on *Paracoccus* phage or a phage genome. Here we describe the first phage isolated from this genus and its genome characteristics. According to the methods of nomenclature of viruses of Kropinski et al. [8] the phage was named vB_PmaS-R3.

## Organism information

### Classification and features

**Fig. 1:**
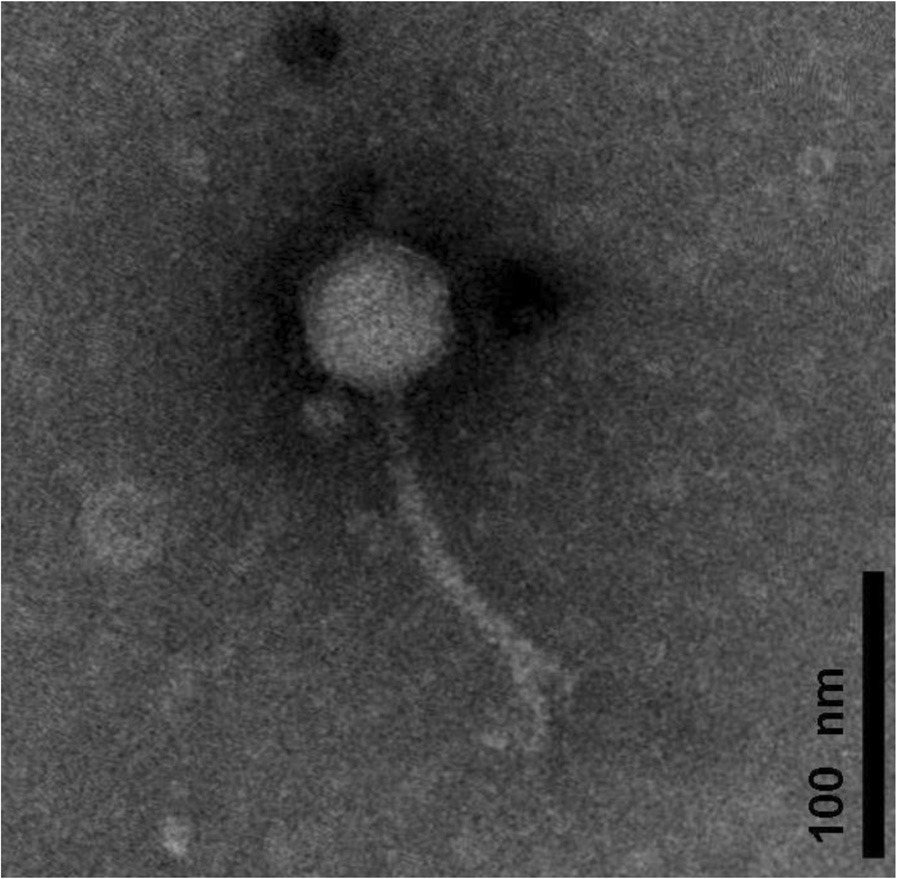
Transmission electron microscopy image of *Paracoccus* phage vB_PmaS-R3

**Fig. 2:**
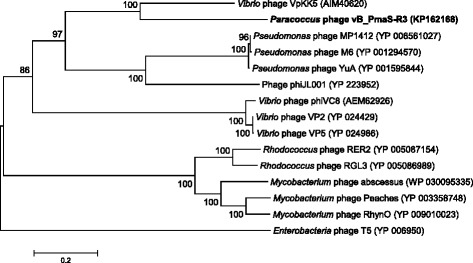
Neighbor-joining phylogenetic tree based on the DNA polymerase amino acid sequences of vB_PmaS-R3 and 14 siphophages. *Enterobacteria* phage T5 was used to root the tree. Bootstrap percentage analyses were based on 1000 replications. Bar, 0.2 substitutions per amino acid position

The host of phage vB_PmaS-R3 *Paracoccus marcusii* strain JL-65 was isolated from the North Pacific Ocean by Du, et al. [6, 9]. Phage vB_PmaS-R3 was a lytic dsDNA phage isolated from the surface seawater of the South China Sea (22.06° N, 118.44° E) (Table 1). According to the transmission electron microscopy image (Fig. 1), vB_PmaS-R3 is a siphophage with an isometric head (ca. 50 nm in diameter) and a long, flexible, non-contractile tail (ca. 122 nm long and ca. 10 nm wide). Phylogenetic analysis based on DNA polymerase gene sequences revealed that vB_PmaS-R3 clustered with *Vibrio* phage VpKK5, and was also closely related to *Pseudomonas* phages MP1412, M6, YuA and phage phiJL001 (Fig. 2).

**Table 1:** Classification and general features of *Paracoccus marcusii* phage vB_PmaS-R3 [35]

MIGS ID	Property	Term	Evidence code^a^
	Classification	Domain: Viruses, dsDNA viruses, no RNA stage	TAS [8, 9]
		Phylum: unassigned	
		Class: unassigned	
		Order: *Caudovirales*	TAS [8, 9]
		Family: *Siphoviridae*	TAS [8, 9]
		Genus: unassigned	
		Species: unassigned	
		(Type) strain: unassigned	
	Gram stain		
	Virus shape	Icosahedral	IDA
	Motility		
	Sporulation		
	Temperature range		
	Optimum temperature		
	pH range; Optimum		
	Carbon source		
MIGS-6	Habitat	Oceanic	IDA
MIGS-6.3	Salinity		
MIGS-22	Oxygen requirement		
MIGS-15	Biotic relationship	Obligate intracellular parasite of *Paracoccus marcusii* strain JL-65	IDA
MIGS-14	Pathogenicity	Lytic phage of *Paracoccus marcusii* strain JL-65	IDA
MIGS-4	Geographic location	South China Sea	IDA
MIGS-5	Sample collection	August, 2008	IDA
MIGS-4.1	Latitude	22.06° N	IDA
MIGS-4.2	Longitude	118.44° E	IDA
MIGS-4.4	Altitude	unknown	

^a^Evidence codes - *IDA* Inferred from direct assay, *TAS* traceable author statement (i.e., a direct report exists in the literature). The evidence codes are the Gene Ontology project [36]

## Genome sequencing information

### Genome project history

Phage vB_PmaS-R3 is the first phage isolated from the genus *Paracoccus*, and is a member of the few known siphophage isolates from the marine environment [10]. Genome sequencing of vB_PmaS-R3 would provide a better understanding of the gene diversity of siphophages in the ocean. The genome project is deposited in GOLD and IMG system. The genome sequence and annotation are available in GenBank (KP162168). A summary of the project information is presented in Table 2.

**Table 2:** Project information

MIGS ID	Property	Term
MIGS-31	Finishing quality	Complete
MIGS-28	Libraries used	454 shotgun library
MIGS-29	Sequencing platforms	454 GS FLX
MIGS-31.2	Fold coverage	100-folds
MIGS-30	Assemblers	GS de novo Assembler software
MIGS-32	Gene calling method	GeneMark.hmm, ORF Finder, RAST server version 2.0, IMG-ER platform
	Locus Tag	NJ83
	Genbank ID	KP162168
	Genbank Date of Release	Jan 18, 2015
	GOLD ID	Gp0109426
	BIOPROJECT	PRJNA263671
MIGS-13	Source Material Identifier	SAMN03104886
	Project relevance	Exploring the marine phage genome

### Growth conditions and genomic DNA preparation

Phage vB_PmaS-R3 was isolated from surface seawater of the South China Sea. The seawater was filtered through a 0.22-μm polycarbonate membrane filter (Millipore, Bedford, MA, USA) and stored at 4 °C. The host strain was cultured at 28 °C using modified rich organic medium (1 g l^−1^ yeast extract, 1 g l^−1^ peptone, 1 l artificial seawater) as described by Yurkov et al. [11]. Two milliliters of seawater filtrate were added to 20 ml exponentially growing bacterial cultures (OD_600_ = 0.2) and incubated for 48 h. Cultures were then centrifuged at 10,000 × *g* for 10 min. The supernatants were collected and filtered through 0.22-μm filters to remove bacterial cells. One hundred microliters of the filtrate were added to 1 ml exponentially growing bacterial culture to perform plaque assays according to the method described by Suttle and Chen [12]. The phage isolate was then purified five times using the plaque assay method. For phage DNA preparation, phage suspension was added to 1 l host culture (OD_600_ = 0.2–0.3) with a multiplicity of infection of 0.01 and incubated overnight at 28 °C, with shaking at 180 rpm. Phage particles in the lysates were harvested and purified using the method described by Chen et al. [13] with some modifications as follows. The lysates were added with 2 mg of both DNase I and RNase A, and incubated at room temperature for 1 h. After the digestion, the NaCl concentration of the lysates was adjusted to 1 M. Then the lysates were incubated on ice for 1 h. The remaining cells and debris were removed by centrifugation at 10,000 × *g* for 10 min followed by filtration through 0.22-μm filters. Polyethylene glycol 8000 was added to the filtrate to a final concentration of 100 g l^−1^ and incubated at 4 °C for 2 d. The phage particles were precipitated by centrifugation at 10,000 × *g* for 80 min, resuspended in 6 ml TM buffer (20 mmol l^−1^ Tris–HCl, 10 mmol l^−1^ MgCl_2_, pH 7.4) and then incubated overnight at 4 °C. CsCl_2_ was added to the phage suspensions to a final concentration of 0.75 g ml^−1^ and the mixture was centrifuged at 200,000 × *g* for 24 h in a S55S-1096 rotor (Hitachi, Tokyo, Japan) using a Himac CS 150 GXL microultracentrifuge (Hitachi, Tokyo, Japan). The visible viral bands were extracted and dialyzed twice in TM buffer overnight at 4 °C. Genomic DNA was extracted according to the method described by Chen et al. [13].

### Genome sequencing and assembly

Genome sequencing and assembly were performed at the Chinese National Human Genome Center, Shanghai. The genome of vB_PmaS-R3 was sequenced using massively parallel pyrosequencing technology (454 GS FLX) [14]. Library construction and sequencing were performed following the manufacturer’s instructions. A total of 15,070 reads with average length of 300 bp were obtained, covering the genome 100-fold. Assembly was performed using the GS *de novo* Assembler software [15] and produced 13 contigs ranging from 975 bp to 14210 bp. Relationships between the contigs were determined by multiplex PCR [16]. Gaps were then filled in by sequencing the PCR products using ABI 3730xl capillary sequencers. Finally, the sequences were assembled using the Phred, Phrap and Consed software packages [17], and low quality regions of the genome were resequenced.

### Genome annotation

Open reading frames (ORFs) were identified by combining results from the GeneMark.hmm 2.0 gene prediction program with heuristic models [18], the ORF Finder [19], the RAST server version 2.0 [20], and the Integrated Microbial Genomes-Expert Review platform [21]. Each translated ORF was blasted against the NCBI non-redundant protein database and the KEGG protein database using BLASTP [22]. The protein domains and COG [23] assignment were predicted by RPS-BLAST against the NCBI CDD library [24].

## Genome properties

**Fig. 3:**
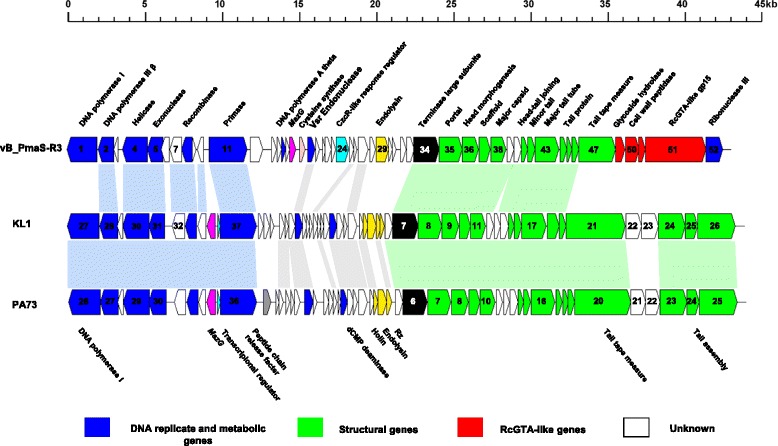
Genome map of *Paracoccus* phage vB_PmaS-R3 and comparative genomic analysis with *Burkholderia* phage KL1 and *Pseudomonas* phage PA73

The properties and statistics of the genome are summarized in Tables 3 and 4. Phage vB_PmaS-R3 has a circular double-stranded DNA genome with a length of 42,093 bp and a G + C content of 56.37 % (Table 3). Fifty-two ORFs were predicted from the genome with a coding efficiency of 92.79 %. Among the 52 ORFs, 11 had no matches in the NCBI non-redundant protein database, 33 were homologous to genes identified in phages, while seven were only found in bacteria. Strikingly, one (ORF30) was found to be closely related to a gene identified from an algal virus, *Dunaliella viridis* virus SI2 (Additional file 1: Table S1). Of the 44 ORFs that have matches in the NCBI database, 28 were assigned to putative functions (Fig. 3, Additional file 1: Table S1). The distribution of genes into COG categories is presented in Table 4.

**Table 3:** Genome statistics

Attribute	Value	% of total^a^
Genome size (bp)	42,093	100.00
DNA Coding (bp)	39,059	92.79
DNA G + C (bp)	23,727	56.37
DNA scaffolds	1	
Total genes	52	100.00
Protein coding genes	52	100.00
RNA genes	0	0.00
Pseudo genes	0	0.00
Genes in internal clusters	3	5.77
Genes with function prediction	28	53.85
Genes assigned to COGs	5	9.62
Genes with Pfam domains	15	28.85
Genes with signal peptides	1	1.92
Genes with transmembrane helices	7	13.46
CRISPR repeats	0	0.00

^a^The total is based on either the size of the genome in base pairs or the total number of protein coding genes in the annotated genome

**Table 4:** Number of genes associated with the 25 general COG functional categories

Code	Value	% of total^a^	Description
J	0	0	Translation, ribosomal structure and biogenesis
A	0	0	RNA processing and modification
K	1	1.92	Transcription
L	3	5.77	Replication, recombination and repair
B	0	0	Chromatin structure and dynamics
D	0	0	Cell cycle control, Cell division, chromosome partitioning
V	0	0	Defense mechanisms
T	0	0	Signal transduction mechanisms
M	0	0	Cell wall/membrane biogenesis
N	0	0	Cell motility
U	0	0	Intracellular trafficking and secretion
O	0	0	Posttranslational modification, protein turnover, chaperones
C	0	0	Energy production and conversion
G	0	0	Carbohydrate transport and metabolism
E	0	0	Amino acid transport and metabolism
F	0	0	Nucleotide transport and metabolism
H	0	0	Coenzyme transport and metabolism
I	0	0	Lipid transport and metabolism
P	0	0	Inorganic ion transport and metabolism
Q	0	0	Secondary metabolites biosynthesis, transport and catabolism
R	0	0	General function prediction only
S	2	3.85	Function unknown
-	46	88.46	Not in COGs

^a^The total is based on the total number of protein coding genes in the annotated genome

## Insights from the genome sequence

### Comparative genomics

According to the BLASTP results, many ORFs in vB_PmaS-R3 genome are homologous to those predicted from the genomes of two siphophages *Burkholderia* phage KL1 (KL1) and *Pseudomonas* phage PA73 (PA73) [25, 26]. Phage vB_PmaS-R3 is genomically similar to these two phages with respect to the genome length (42,832 bp and 42,999 bp for KL1 and PA73, respectively), G + C content (54.6 and 53.6 % for KL1 and PA73) and number of predicted ORFs (55 and 52 for KL1 and PA73). Of the 52 ORFs of the vB_PmaS-R3 genome, 22 are found to be homologous to mutual genes in KL1 and PA73, with one additional gene homologous to a KL1 gene (Fig. 3). These genes are mainly distributed in the DNA metabolism and morphogenesis encoding regions, which are deemed the core parts of the genome. The vB_PmaS-R3 genome could be divided into three modules: genes related to DNA replication and metabolism, regulatory genes, and structure forming genes. In the DNA replication and metabolic module, of the seven ORFs with putative functions, six are homologues to genes of KL1 and PA73. However, the DNA polymerase (ORF1) in the vB_PmaS-R3 genome is phylogenetically distant from those in the KL1 and PA73 genomes. Phage vB_PmaS-R3 has DNA polymerase type I, which belongs to DNA polymerase A family, whereas the DNA polymerases in KL1 and PA73 are members of the polymerase B family. Phage vB_PmaS-R3 shares similar structural genes with KL1 and PA73 (Fig. 3). However, the tail tape-measure gene in vB_PmaS-R3 is very different from those in KL1 and PA73. Furthermore, KL1 and PA73 have three tail assembly genes following the tail tape- measure gene, while in the vB_PmaS-R3 genome, the tail tape-measure gene is followed by four gene transfer-agent like genes instead (Fig. 3). The lysis gene of vB_PmaS-R3 is a single L-alanoyl-D-glutamate peptidase-like gene [27], while in KL1 and PA73, a combination of four and three genes function as the lysis gene, respectively.

### Auxiliary metabolic genes

AMGs are phage-encoded metabolic genes that could putatively regulate the host metabolism and represent phage genomic adaptations to the host and environment [28]. In the vB_PmaS-R3 genome, ORF17 is predicted to encode a *MazG* domain-containing protein, which is frequently described as an AMG. Recently, phage-encoded *MazG* has received much attention. It is widely distributed in marine cyanophages [29, 30] and can also be found in viruses isolated from heterotrophic bacteria, including marine and non-marine bacteria [25, 31–35]. It is suggested that the expression of phage *MazG* could help to maintain the metabolism of starved infected host cells in nutrient-limited conditions and to optimize the production of progeny phage.

## Conclusions

Phage vB_PmaS-R3 represents the first marine phage isolate infecting *Paracoccus* spp. According to the genome analysis, this phage is most likely a member of the viral family *Siphoviridae.* The genome is modularly organized, and shows synteny with the genomes of *Burkholderia* phage KL1 and *Pseudomonas* phage PA73 in the DNA metabolism and morphogenesis modules. The DNA polymerase, tail tape-measure and lysis genes reveal the difference between phage vB_PmaS-R3 and phages KL1 and PA73. The *MazG* gene may have the potential to benefit phage propagation of phage vB_PmaS-R3 in its nutrient-limited environment. The genome analysis of phage vB_PmaS-R3 enriches our knowledge of the evolutionary history of marine phages and ecological understanding of phage adaption to the environment.

## Additional file

Additional file 1: Table S1.
*Paracoccus* phage vB_PmaS-R3 gene annotations. (DOCX 18 kb)

## Abbreviations

AMG: Auxiliary metabolic gene
IMG: Integrated microbial genomes
ORF: Open reading frame
RAST: Rapid annotation using subsystem technology
