# Supplementary material for: Complete genome sequence of Paracoccus marcusii phage vB_PmaS-R3 isolated from the South China Sea
Source: Stand Genomic Sci. 2015 Nov 10;10:94. doi: 10.1186/s40793-015-0089-7 (PMC4641407; doi:10.1186/s40793-015-0089-7)
Supplement: Additional file 1: Table S1. — Paracoccus phage vB_PmaS-R3 gene annotations. (DOCX 18 kb) [file 40793_2015_89_MOESM1_ESM.docx]

Supplementary table

Additional ﬁle 1 to: “Complete genome sequence of *Paracoccus marcusii* phage vB_PmaS-R3 isolated from the South China Sea”

Authors: Yongle Xu, Rui Zhang^*^ , Nianzhi Jiao^*^

*Corresponding author: Rui Zhang ([ruizhang@xmu.edu.cn](mailto:ruizhang@xmu.edu.cn)), Nianzhi Jiao ([jiao@xmu.edu.cn](mailto:jiao@xmu.edu.cn))

**Additional file 1: Table S1.** *Paracoccus* phage vB_PmaS-R3 gene annotations.

| **ORF no.** | **Protein size (aa)** | **Significant hit (Organism)** | **E-value** | **Putative function** | **% aa identity** |
| --- | --- | --- | --- | --- | --- |
| 1 | 672 | hypothetical protein (*Vibrio* phage VpKK5) | 0 | DNA polymerase I | 48% |
| 2 | 337 | DNA polymerase III beta subunit (*Burkholderia* phage KL1) | 3.00E-65 | DNA polymerase III beta subunit | 38% |
| 3 | 102 | hypothetical protein (*Sinorhizobium* sp. CCBAU 05631) | 2.00E-13 | hypothetical protein | 47% |
| 4 | 541 | hypothetical protein ORF029 (*Pseudomonas* phage 73) | 0 | superfamily II helicase | 52% |
| 5 | 325 | exonuclease (*Burkholderia* phage KL1) | 2.00E-104 | exonuclease | 50% |
| 6 | 168 | hypothetical protein (*Thioalkalivibrio* sp. ALE19) | 1.00E-18 | hypothetical protein | 39% |
| 7 | 243 | hypothetical protein (*Vibrio* phage VpKK5) | 3.00E-42 | hypothetical protein | 52% |
| 8 | 227 | hypothetical protein ORF032 (*Pseudomonas* phage 73) | 3.00E-81 | ATPase AAA | 52% |
| 10 | 141 | hypothetical protein IME_AB3_13 (*Acinetobacter* phage IME_AB3) | 2.00E-18 | hypothetical protein | 34% |
| 11 | 805 | replicative primase/helicase (*Pseudomonas* phage vB_Pae-Kakheti25) | 0 | primase | 47% |
| 15 | 103 |  |  | DNA_pol_A_theta |  |
| 17 | 142 | putative nucleoside triphosphate pyrophosphohydrolase (*Rhizobium* phage vB_RglS_P106B) | 2.00E-35 | MazG family pyrophosphatase | 49% |
| 18 | 113 | hypothetical protein (*Algiphilus aromaticivorans*) | 8.00E-13 | putative cysteine synthase | 46% |
| 19 | 171 | Vsr endonuclease (*Burkholderia* phage KL1) | 9.00E-34 | Vsr endonuclease | 46% |
| 20 | 80 | hypothetical protein CbK_gp054 (*Caulobacter* phage phiCbK) | 2.00E-10 | hypothetical protein | 46% |
| 21 | 77 | hypothetical protein MP1412_28 (*Pseudomonas* phage MP1412) | 3.00E-09 | hypothetical protein | 42% |
| 24 | 267 | hypothetical protein (*Sinorhizobium* *medicae*) | 2.00E-15 | CzcR-like response regulator | 48% |
| 25 | 91 | hypothetical protein KL1_00048 (*Burkholderia* phage KL1) | 1.00E-05 | hypothetical protein | 36% |
| 26 | 76 | hypothetical protein RDJLphi1_gp10 (*Roseobacter* phage RDJL Phi 1) | 3.00E-09 | hypothetical protein | 48% |
| 27 | 217 | hypothetical protein IME_AB3_46 (*Acinetobacter* phage IME_AB3) | 2.00E-35 | hypothetical protein | 42% |
| 29 | 271 | hypothetical protein (*Rhodobacter* *sphaeroides*) | 6.00E-80 | endolysin | 51% |

**Table S1.** *Paracoccus* phage vB_PmaS-R3 gene annotations (Continued)

| 30 | 79 | hypothetical protein DVVG_00017 (*Dunaliella* *viridis* virus SI2) | 8.00E-13 | hypothetical protein | 43% |
| --- | --- | --- | --- | --- | --- |
| 32 | 118 | hypothetical protein (*Ochrobactrum* *anthropi*) | 3.00E-14 | D-amino acid aminotransferase | 42% |
| 34 | 540 | terminase large subunit (*Burkholderia* phage KL1) | 0 | terminase large subunit | 64% |
| 35 | 492 | portal protein (*Pseudomonas* phage vB_PaeS_SCH_Ab26) | 1.00E-161 | portal protein | 54% |
| 36 | 346 | head morphogenesis protein (*Burkholderia* phage KL1) | 8.00E-49 | head morphogenesis protein | 30% |
| 37 | 247 | scaffold protein (*Pseudomonas phage* vB_PaeS_SCH_Ab26) | 1.00E-71 | scaffold protein | 55% |
| 38 | 320 | hypothetical protein ORF010 (*Pseudomonas* phage 73) | 6.00E-150 | major capsid protein | 66% |
| 39 | 149 | hypothetical protein ORF011 (*Pseudomonas* phage 73) | 5.00E-08 | hypothetical protein | 39% |
| 40 | 159 | virion protein (Pseudomonas phage vB_PaeS_SCH_Ab26) | 4.00E-18 | hypothetical protein | 34% |
| 41 | 131 | virion protein (*Pseudomonas* phage vB_Pae-Kakheti25) | 4.00E-17 | head-tail joining protein | 37% |
| 42 | 155 | virion protein (*Pseudomonas* phage vB_PaeS_SCH_Ab26) | 7.00E-20 | minor tail protein | 31% |
| 43 | 512 | major tail tube protein (*Pseudomonas* phage vB_PaeS_SCH_Ab26) | 9.00E-164 | major tail tube protein | 53% |
| 44 | 148 | tail chaperonin (*Pseudomonas* phage vB_PaeS_SCH_Ab26) | 2.00E-15 | tail protein | 36% |
| 45 | 106 | tail chaperonin (*Pseudomonas* phage vB_PaeS_SCH_Ab26) | 2.00E-17 | tail chaperonin | 45% |
| 46 | 148 | hypothetical protein ORF019 (*Pseudomonas* phage 73) | 7.00E-23 | hypothetical protein | 45% |
| 47 | 784 | lambda phage tail tape-measure protein (*Ruegeria* *lacuscaerulensis*) | 4.00E-53 | phage tail tape-measure protein | 38% |
| 48 | 210 | glycoside hydrolase family 24 (*Celeribacter* *indicus*) | 8.00E-80 | glycoside hydrolase family protein | 58% |
| 49 | 295 | hypothetical protein (*Brucella abortus*) | 3.00E-98 | gene transfer agent gp13 | 49% |
| 50 | 144 | phage cell wall peptidase, NlpC/P60 family (*Methylobacterium* *aquaticum*) | 1.00E-35 | phage cell wall peptidase | 48% |
| 51 | 1292 | hypothetical protein (*Methylocystis* sp. LW5) | 0 | gene transfer agent gp15 | 41% |
| 52 | 359 | hypothetical protein (*Afifella pfennigii*) | 7.00E-71 | ribonuclease III | 40% |
